# Supplementary material for: Mechanisms governing the pioneering and redistribution capabilities of the non-classical pioneer PU.1
Source: Nat Commun. 2020 Jan 21;11:402. doi: 10.1038/s41467-019-13960-2 (PMC6972792; doi:10.1038/s41467-019-13960-2)
Supplement: Supplementary file 7 — Source data [file 41467_2019_13960_MOESM7_ESM.zip › Source_Data/FigureS2/FigureS2B_MotifScanOutput/KmeansCluster5/homerResults.html]

/misc/data/analysis/project\_PU1/CTV1/motifs/basic/PU1\_Kmeans14.5/final - Homer de novo Motif Results


# Homer *de novo* Motif Results (/misc/data/analysis/project\_PU1/CTV1/motifs/basic/PU1\_Kmeans14.5/final)

Known Motif Enrichment Results  
Gene Ontology Enrichment Results  
If Homer is having trouble matching a motif to a known motif, try copy/pasting the matrix file into
STAMP  
More information on motif finding results: HOMER
| Description of Results
| Tips
  
Total target sequences = 3980  
Total background sequences = 42833  
\* - possible false positive  

|  |  |  |  |  |  |  |  |  |
| --- | --- | --- | --- | --- | --- | --- | --- | --- |
| Rank | Motif | P-value | log P-pvalue | % of Targets | % of Background | STD(Bg STD) | Best Match/Details | Motif File |
| 1 | A T G C C G T A A T G C C G A T A C G T A G T C A G T C A G C T A G T C G A C T G A C T G A C T | 1e-2011 | -4.631e+03 | 82.41% | 11.34% | 31.7bp (81.9bp) | SpiB(ETS)/OCILY3-SPIB-ChIP-Seq(GSE56857)/Homer(0.965) More Information \| Similar Motifs Found | motif file (matrix) |
| 2 | G C T A C T G A T C A G T A G C T C A G T C A G C T G A G T C A | 1e-527 | -1.213e+03 | 63.79% | 24.83% | 46.5bp (76.0bp) | SpiB(ETS)/OCILY3-SPIB-ChIP-Seq(GSE56857)/Homer(0.764) More Information \| Similar Motifs Found | motif file (matrix) |
| 3 | A T C G C T G A A C T G C G T A C G T A A C T G A C G T C T A G | 1e-344 | -7.931e+02 | 27.81% | 6.16% | 44.5bp (77.6bp) | SpiB(ETS)/OCILY3-SPIB-ChIP-Seq(GSE56857)/Homer(0.747) More Information \| Similar Motifs Found | motif file (matrix) |
| 4 | A T G C A C G T C G A T A G T C A C G T C A G T A G T C A C G T | 1e-263 | -6.059e+02 | 45.13% | 19.49% | 54.8bp (76.9bp) | Unknown4/Arabidopsis-Promoters/Homer(0.772) More Information \| Similar Motifs Found | motif file (matrix) |
| 5 | A T C G A G C T C G A T G T A C A T G C G C A T A T G C G T A C G A T C | 1e-253 | -5.847e+02 | 39.02% | 15.38% | 50.9bp (74.6bp) | MYB98(MYB)/col-MYB98-DAP-Seq(GSE60143)/Homer(0.756) More Information \| Similar Motifs Found | motif file (matrix) |
| 6 | C T G A A T G C C G A T A C G T G T A C A C G T T A C G G A T C C G A T C A G T A G C T A G C T | 1e-175 | -4.044e+02 | 14.95% | 3.28% | 44.3bp (75.2bp) | SpiB(ETS)/OCILY3-SPIB-ChIP-Seq(GSE56857)/Homer(0.769) More Information \| Similar Motifs Found | motif file (matrix) |
| 7 | T A G C G C T A A T C G C G A T G C A T G T C A A G T C G A C T A G C T A T G C G T A C | 1e-105 | -2.430e+02 | 12.19% | 3.51% | 49.1bp (77.5bp) | PB0012.1\_Elf3\_1/Jaspar(0.667) More Information \| Similar Motifs Found | motif file (matrix) |
| 8 | C G T A A G T C C T G A A C T G A T C G C G T A C G A T A C T G | 1e-89 | -2.062e+02 | 19.42% | 8.57% | 55.4bp (69.8bp) | Ets1-distal(ETS)/CD4+-PolII-ChIP-Seq(Barski\_et\_al.)/Homer(0.820) More Information \| Similar Motifs Found | motif file (matrix) |
| 9 | A C G T T G A C A C G T G A T C C T G A G A C T C T A G C G T A A C T G C T G A C G T A G T A C G C A T A T C G | 1e-80 | -1.862e+02 | 4.95% | 0.69% | 42.7bp (75.1bp) | ZBTB33(Zf)/GM12878-ZBTB33-ChIP-Seq(GSE32465)/Homer(0.726) More Information \| Similar Motifs Found | motif file (matrix) |
| 10 | T C G A A C G T A C T G C T G A A G C T C G A T A C T G C G A T C T G A C G T A T A C G C G A T C A G T | 1e-80 | -1.857e+02 | 4.27% | 0.48% | 66.6bp (73.2bp) | EXC-7(RRM)/Caenorhabditis\_elegans-RNCMPT00014-PBM/HughesRNA(0.618) More Information \| Similar Motifs Found | motif file (matrix) |
| 11 | C G T A A T C G A C G T C A T G C G T A C A T G A C G T G C A T A G T C C G A T A G T C T C G A | 1e-76 | -1.770e+02 | 3.42% | 0.27% | 53.2bp (74.1bp) | STZ(C2H2)/colamp-STZ-DAP-Seq(GSE60143)/Homer(0.657) More Information \| Similar Motifs Found | motif file (matrix) |
| 12 | G T A C A G C T A G T C G A T C G C A T A G T C G T A C C G A T A T G C G A C T A C G T A T G C G T A C A G C T | 1e-76 | -1.766e+02 | 6.18% | 1.24% | 49.5bp (92.9bp) | ZNF263/MA0528.1/Jaspar(0.708) More Information \| Similar Motifs Found | motif file (matrix) |
| 13 | C T A G T C G A C G A T T G C A C T A G A C G T C A T G G C T A C A T G G C A T | 1e-68 | -1.581e+02 | 5.03% | 0.90% | 40.1bp (71.7bp) | At5g04390(C2H2)/col200-At5g04390-DAP-Seq(GSE60143)/Homer(0.732) More Information \| Similar Motifs Found | motif file (matrix) |
| 14 | T G C A T G C A A G C T T A G C C T G A C G A T C T A G C A T G G T A C C T A G A T C G G T C A C G T A | 1e-66 | -1.531e+02 | 4.65% | 0.78% | 67.4bp (67.6bp) | YY2/MA0748.1/Jaspar(0.678) More Information \| Similar Motifs Found | motif file (matrix) |
| 15 | C T G A G C T A T C A G C G T A C A T G T A G C C A G T A C T G C T A G A G C T A C G T T A C G A G C T A C G T | 1e-65 | -1.504e+02 | 3.74% | 0.48% | 51.1bp (68.6bp) | GATA11/MA1014.1/Jaspar(0.627) More Information \| Similar Motifs Found | motif file (matrix) |
| 16 | A G T C A G T C A G T C A G T C C T G A A C G T A C T G C G T A A C G T A G T C A G T C C G T A C T G A A C G T | 1e-59 | -1.375e+02 | 3.07% | 0.32% | 77.0bp (67.1bp) | Atf4(bZIP)/MEF-Atf4-ChIP-Seq(GSE35681)/Homer(0.639) More Information \| Similar Motifs Found | motif file (matrix) |
| 17 | C A G T A C T G G A C T G A C T G C A T T C G A T C G A T C G A C G T A T C A G C G A T T C A G G C A T C A T G | 1e-57 | -1.324e+02 | 3.17% | 0.38% | 32.7bp (66.7bp) | CHR(?)/Hela-CellCycle-Expression/Homer(0.644) More Information \| Similar Motifs Found | motif file (matrix) |
| 18 | A C T G A C G T C T A G C T G A A G C T A C G T C A T G T C A G C G T A C G A T G A T C C G T A | 1e-55 | -1.268e+02 | 2.16% | 0.13% | 77.7bp (73.4bp) | PH0014.1\_Cphx/Jaspar(0.831) More Information \| Similar Motifs Found | motif file (matrix) |
| 19 | A G C T A C G T A T G C A G T C C G A T T G A C G C T A T G C A G C T A G C A T | 1e-49 | -1.147e+02 | 4.30% | 0.93% | 52.0bp (67.5bp) | SPT2(MacIsaac)/Yeast(0.736) More Information \| Similar Motifs Found | motif file (matrix) |
| 20 | C T A G C A T G G C A T C A G T C T A G A G C T G A C T G A C T C T G A C T G A G C T A T C G A | 1e-47 | -1.101e+02 | 3.99% | 0.83% | 36.3bp (72.4bp) | TCX2(CPP)/colamp-TCX2-DAP-Seq(GSE60143)/Homer(0.683) More Information \| Similar Motifs Found | motif file (matrix) |
| 21 | A C T G A T C G C G T A C T G A T A C G A G C T C G T A C T G A T C A G A G T C G T C A | 1e-47 | -1.098e+02 | 6.58% | 2.14% | 53.1bp (76.6bp) | AT5G25475(ABI3VP1)/col-AT5G25475-DAP-Seq(GSE60143)/Homer(0.691) More Information \| Similar Motifs Found | motif file (matrix) |
| 22 | G A T C G C T A T C G A G A T C A G T C C G T A A G T C T G C A G C A T | 1e-46 | -1.065e+02 | 23.27% | 14.27% | 56.6bp (67.7bp) | RUNX1(Runt)/Jurkat-RUNX1-ChIP-Seq(GSE29180)/Homer(0.858) More Information \| Similar Motifs Found | motif file (matrix) |
| 23 | C T A G C T A G A C T G C T G A C T G A T C A G A G T C G A T C G A C T A G T C C G T A A C T G C T A G C T G A | 1e-43 | -1.005e+02 | 3.09% | 0.53% | 70.4bp (67.7bp) | GCR2/MA0305.1/Jaspar(0.675) More Information \| Similar Motifs Found | motif file (matrix) |
| 24 | A C G T A C T G A G T C A G C T T A C G G C A T G A C T G A T C C G A T A G T C C T G A | 1e-43 | -1.001e+02 | 4.87% | 1.35% | 53.6bp (65.8bp) | YBX2(CSD)/Homo\_sapiens-RNCMPT00084-PBM/HughesRNA(0.726) More Information \| Similar Motifs Found | motif file (matrix) |
| 25 | G T A C G C A T G C A T A G T C G A T C A T G C A G T C A C G T A G C T A C G T C T A G T G A C A G T C A G C T | 1e-42 | -9.784e+01 | 2.04% | 0.19% | 52.5bp (73.3bp) | Pparg::Rxra/MA0065.2/Jaspar(0.681) More Information \| Similar Motifs Found | motif file (matrix) |
| 26 | C T G A G A T C A T C G G T C A C A T G G C T A G C A T T A G C G C A T | 1e-42 | -9.716e+01 | 4.80% | 1.34% | 39.2bp (72.0bp) | GATA14(C2C2gata)/col-GATA14-DAP-Seq(GSE60143)/Homer(0.857) More Information \| Similar Motifs Found | motif file (matrix) |
| 27 | A C G T G T A C A C G T A C G T G T A C G T C A A G T C C T G A C G A T A C T G C T A G A G C T T C A G T C A G | 1e-40 | -9.237e+01 | 1.71% | 0.13% | 39.9bp (68.4bp) | INO4/INO4\_YPD/4-INO4,37-INO2(Harbison)/Yeast(0.758) More Information \| Similar Motifs Found | motif file (matrix) |
| 28 | C T G A A C T G G T C A C T G A A T C G C G T A C G T A A C T G C T G A C G A T C T A G C T G A C T A G A C T G | 1e-39 | -9.135e+01 | 4.15% | 1.08% | 54.6bp (79.4bp) | TRA2(RRM)/Drosophila\_melanogaster-RNCMPT00078-PBM/HughesRNA(0.688) More Information \| Similar Motifs Found | motif file (matrix) |
| 29 | C T G A A T C G C T A G C T G A C G T A C G T A A G T C A C G T A C G T C G T A | 1e-38 | -8.974e+01 | 3.12% | 0.62% | 72.9bp (69.3bp) | UME1/UME1\_YPD/[](Harbison)/Yeast(0.768) More Information \| Similar Motifs Found | motif file (matrix) |
| 30 | C T A G C A G T G A C T G A C T A G T C A G T C G A T C G A T C A G T C G C A T A C G T T A C G A G T C | 1e-38 | -8.805e+01 | 2.46% | 0.38% | 61.1bp (71.4bp) | MSN4(MacIsaac)/Yeast(0.694) More Information \| Similar Motifs Found | motif file (matrix) |
| 31 | T C G A T A C G C A G T A G C T C T G A G A T C C T G A T C A G C A G T G T A C | 1e-37 | -8.728e+01 | 2.46% | 0.38% | 68.9bp (69.2bp) | PUM(PUF)/Drosophila\_melanogaster-RNCMPT00103-PBM/HughesRNA(0.746) More Information \| Similar Motifs Found | motif file (matrix) |
| 32 | A C T G C G T A A C T G A C T G A C G T A C T G C G T A A G C T A C G T A C T G | 1e-36 | -8.484e+01 | 1.96% | 0.22% | 80.4bp (72.3bp) | Dux/MA0611.1/Jaspar(0.702) More Information \| Similar Motifs Found | motif file (matrix) |
| 33 | C G T A G A C T G A T C C G T A A G C T T C A G A T C G A G T C G C T A T C A G | 1e-36 | -8.298e+01 | 4.15% | 1.17% | 63.6bp (65.5bp) | CHA4(MacIsaac)/Yeast(0.688) More Information \| Similar Motifs Found | motif file (matrix) |
| 34 | C G A T A G C T C G T A T G C A G T C A G C T A A C T G G C A T T A C G A C G T A C T G C G A T C T G A | 1e-34 | -8.024e+01 | 2.74% | 0.54% | 51.4bp (64.7bp) | PAPI(KH)/Drosophila\_melanogaster-RNCMPT00011-PBM/HughesRNA(0.671) More Information \| Similar Motifs Found | motif file (matrix) |
| 35 | A G T C A G T C C A G T C A T G T A C G C G A T C T A G T C A G C T A G C T G A C T A G A C T G C A G T T C A G | 1e-34 | -7.981e+01 | 2.11% | 0.30% | 75.5bp (63.7bp) | GLIS3/MA0737.1/Jaspar(0.674) More Information \| Similar Motifs Found | motif file (matrix) |
| 36 | C T G A A T C G G T C A C T G A T C A G T G C A C G T A T C A G T G A C C G T A A C T G G T C A C T G A A C T G | 1e-32 | -7.589e+01 | 3.29% | 0.82% | 52.9bp (69.6bp) | ETS:E-box(ETS,bHLH)/HPC7-Scl-ChIP-Seq(GSE22178)/Homer(0.638) More Information \| Similar Motifs Found | motif file (matrix) |
| 37 | A G T C A G C T A G T C A C G T A T G C A C G T G T A C C G A T T A G C A G T C G A C T T C A G G T A C | 1e-32 | -7.534e+01 | 2.14% | 0.33% | 36.6bp (81.0bp) | SeqBias: GA-repeat(0.788) More Information \| Similar Motifs Found | motif file (matrix) |
| 38 | C G A T C G T A C T G A G T C A G T C A A T C G A C G T C T A G A C G T C T A G A C G T C T A G A C T G | 1e-29 | -6.820e+01 | 1.58% | 0.18% | 44.3bp (77.2bp) | Rbm38(RRM)/Danio\_rerio-RNCMPT00283-PBM/HughesRNA(0.733) More Information \| Similar Motifs Found | motif file (matrix) |
| 39 | A G C T A G T C C G T A A G T C C T G A C G A T C A T G T C A G A G T C | 1e-28 | -6.465e+01 | 6.91% | 3.15% | 51.0bp (67.9bp) | USF2/MA0526.1/Jaspar(0.892) More Information \| Similar Motifs Found | motif file (matrix) |
| 40 | A C T G G T C A A T C G A T C G C G T A A G C T C T A G C G A T A C T G C A T G | 1e-27 | -6.260e+01 | 6.28% | 2.78% | 53.2bp (86.7bp) | ETS:RUNX(ETS,Runt)/Jurkat-RUNX1-ChIP-Seq(GSE17954)/Homer(0.813) More Information \| Similar Motifs Found | motif file (matrix) |
| 41 | G T C A C T G A C A T G C T A G A C T G T A C G C G T A T C G A A C T G C T G A C T A G C T G A C G T A | 1e-26 | -6.078e+01 | 1.16% | 0.09% | 51.4bp (63.2bp) | Trl/dmmpmm(SeSiMCMC)/fly(0.709) More Information \| Similar Motifs Found | motif file (matrix) |
| 42 | A G T C C G A T A C T G C G T A A G C T A C T G A C T G A C G T A C G T C G A T | 1e-25 | -5.957e+01 | 1.93% | 0.36% | 41.6bp (67.8bp) | YBX1(CSD)/Homo\_sapiens-RNCMPT00116-PBM/HughesRNA(0.693) More Information \| Similar Motifs Found | motif file (matrix) |
| 43 | T G C A C T G A G T C A A T C G A G T C T A C G A G C T C T A G A G C T | 1e-25 | -5.866e+01 | 2.16% | 0.46% | 30.2bp (63.1bp) | Tp\_0225(RRM)/Thalassiosira\_pseudonana-RNCMPT00225-PBM/HughesRNA(0.758) More Information \| Similar Motifs Found | motif file (matrix) |
| 44 | A C G T A T G C G A C T A T G C C A T G G A T C G A C T C G A T T C A G T G A C | 1e-23 | -5.392e+01 | 2.86% | 0.86% | 40.9bp (70.4bp) | IRF8(IRF)/BMDM-IRF8-ChIP-Seq(GSE77884)/Homer(0.630) More Information \| Similar Motifs Found | motif file (matrix) |
| 45 | C T G A A C G T C T G A C T A G T A C G C G T A G A C T C T A G G A C T C A T G A G T C | 1e-22 | -5.184e+01 | 1.88% | 0.40% | 49.4bp (65.5bp) | PB0181.1\_Spdef\_2/Jaspar(0.754) More Information \| Similar Motifs Found | motif file (matrix) |
| 46 | A G T C A G T C A G C T A C T G G T C A A C T G A C T G A G T C A G T C | 1e-20 | -4.762e+01 | 3.42% | 1.26% | 70.7bp (66.7bp) | AP-2gamma(AP2)/MCF7-TFAP2C-ChIP-Seq(GSE21234)/Homer(0.810) More Information \| Similar Motifs Found | motif file (matrix) |
| 47 | C G T A A C T G A T C G A C G T A C G T A G T C A G T C A C G T A G T C C G T A G T C A C G T A C G T A C G T A | 1e-19 | -4.556e+01 | 0.68% | 0.03% | 59.5bp (40.3bp) | SPIB/MA0081.1/Jaspar(0.658) More Information \| Similar Motifs Found | motif file (matrix) |
| 48 | A C T G A C G T C T G A C G T A A G C T C G A T A C T G | 1e-18 | -4.290e+01 | 15.65% | 10.77% | 64.4bp (67.2bp) | YHP1/MA0426.1/Jaspar(0.898) More Information \| Similar Motifs Found | motif file (matrix) |
| 49 | G C A T C G T A G C A T C T G A G C T A G T C A C G T A A T G C A G T C C T G A A C G T G T A C | 1e-17 | -4.128e+01 | 1.26% | 0.22% | 37.0bp (58.6bp) | grh/dmmpmm(Pollard)/fly(0.710) More Information \| Similar Motifs Found | motif file (matrix) |
| 50 | C A T G T A C G A C T G G T C A C A T G A C T G T A G C A T G C A C T G A G T C | 1e-16 | -3.877e+01 | 1.71% | 0.44% | 71.4bp (66.8bp) | HNRNPH2(RRM)/Homo\_sapiens-RNCMPT00160-PBM/HughesRNA(0.656) More Information \| Similar Motifs Found | motif file (matrix) |
| 51 | A C T G A C G T C T A G C G T A C G T A A C T G C G T A C T G A A C T G A C T G A C G T | 1e-16 | -3.738e+01 | 0.80% | 0.09% | 41.6bp (65.5bp) | TRA2(RRM)/Drosophila\_melanogaster-RNCMPT00078-PBM/HughesRNA(0.750) More Information \| Similar Motifs Found | motif file (matrix) |
| 52 | A C T G A C T G C G T A A C T G A C T G C G T A A C T G A C T G C G T A | 1e-16 | -3.737e+01 | 4.85% | 2.42% | 53.4bp (89.7bp) | SRSF1(RRM)/Homo\_sapiens-RNCMPT00163-PBM/HughesRNA(0.835) More Information \| Similar Motifs Found | motif file (matrix) |
| 53 | A G C T C G A T A G T C G T A C C G A T A G T C G T A C C T G A C G A T G T A C C G T A | 1e-16 | -3.690e+01 | 2.24% | 0.74% | 59.0bp (65.3bp) | HAP3(CCAATHAP3)/col-HAP3-DAP-Seq(GSE60143)/Homer(0.716) More Information \| Similar Motifs Found | motif file (matrix) |
| 54 | C G T A A G T C A G T C C G T A A C T G C G T A A C G T | 1e-15 | -3.656e+01 | 4.45% | 2.16% | 50.7bp (66.3bp) | CG14718(RRM)/Drosophila\_melanogaster-RNCMPT00006-PBM/HughesRNA(0.833) More Information \| Similar Motifs Found | motif file (matrix) |
| 55 | A G T C A T C G A C T G A C G T C T A G C T A G A C T G G T C A | 1e-15 | -3.459e+01 | 10.60% | 6.98% | 60.3bp (66.1bp) | STB5/MA0392.1/Jaspar(0.783) More Information \| Similar Motifs Found | motif file (matrix) |
| 56 | A T C G G T C A G C T A C G A T C A G T C A T G A C G T G T A C C T A G G C A T | 1e-12 | -2.985e+01 | 1.23% | 0.30% | 59.1bp (69.5bp) | GFY(?)/Promoter/Homer(0.735) More Information \| Similar Motifs Found | motif file (matrix) |
